# Active learning for an evidence-based veterinary medicine course during COVID-19

**DOI:** 10.3389/fvets.2022.953687

**Published:** 2022-07-22

**Authors:** Sophie St-Hilaire, Omid Nekouei, Rebecca S. V. Parkes, Sarah M. Rosanowski

**Affiliations:** ^1^Department of Infectious Diseases and Public Health, Jockey Club College of Veterinary Medicine and Life Sciences, City University of Hong Kong, Kowloon, Hong Kong SAR, China; ^2^Department of Veterinary Clinical Sciences, Jockey Club College of Veterinary Medicine and Life Sciences, City University of Hong Kong, Kowloon, Hong Kong SAR, China; ^3^Grasslands Research Center, Ag Research Limited, Palmerston North, New Zealand

**Keywords:** evidence-based veterinary medicine, veterinary education, epidemiology, active learning, student engagement

## Abstract

Epidemiology is often a challenging course that is not well appreciated by many students learning veterinary medicine. The curriculum for this topic can sometimes be dry, difficult for students to contextualize, and heavy with statistics and mathematical concepts. We incorporated the concepts of epidemiology that are most important for practicing veterinarians and combined these with evidence-based veterinary medicine principles to create a practical course for second-year undergraduate veterinary students. We share the structure of our course and the different learning components, which also included incorporating graduate student mentors for journal clubs and an assignment that culminated in some students publishing their review findings. Anecdotal responses from students suggest they enjoyed the course and learned skills they felt would be useful in veterinary practice to help them make evidence-based clinical decisions.

## Introduction

Veterinary education is evolving. In the last 20 years, many schools have shifted their focus to competency-based professional education (1, 2). Achieving competencies requires complementary teaching styles that blend didactic information delivery, problem-based active learning initiatives, clinical critical thinking, and practical skills exercises (3). Many veterinary schools around the world are shifting to a less didactic approach of teaching and more problem-based to foster deep learning and problem-solving skills. Deeper learning approaches are not only associated with ‘higher quality' learning, but also lead to higher grades (4, 5), and encourage the development of skills that assist with clinical reasoning. Evidence-based veterinary medicine (EBVM), which promotes the use of scientific evidence and clinical expertise to make medical decisions, plays an important role in developing these skills, as the appraisal of evidence is an essential basic skill that underpins clinical reasoning (6).

In addition to addressing the teaching strategies used to promote competency-based education, assessments need to be considered. Practical skills and rational problem-solving skills should be incorporated in the assessment process. However, assessing multi-level cognitive competencies is more complicated than basic knowledge assessments. To further compound the difficulty, since 2020, many teaching and assessment activities in veterinary schools around the world have been conducted fully or at least partially online due to the COVID-19 pandemic (7, 8).

Some courses are more conducive to problem-based learning and other active learning exercises than others. For example, physiology or medicine courses can use case-based teaching relatively easily (9). In the case of EBVM, epidemiology or biostatistics courses, which tend to be taught more passively, it can be challenging to design active learning exercises, especially when classes are online. The use of a knowledge summary and group work to teach EBVM has recently been documented in veterinary nursing (10), and individually developed ‘critically appraised topics' have been used in veterinary medicine (11).

At the new college of veterinary medicine at City University of Hong Kong, we delivered a second- year course in EBVM as part of the undergraduate veterinary medicine curriculum. The overarching goal of this course is to teach students how to make evidence-based decisions in clinical practice and to foster deep learning and critical thinking skills. This course combined the basic principles of epidemiology such as sensitivity and specificity of diagnostic tests, how to interpret clinical presentations and diagnostic tests to make clinical decisions, how to search for literature to answer clinical questions, and how to interpret and how to weigh the value of study results based on types of studies conducted, outcomes measured, and potential biases. In this paper, we describe how we created different levels of active learning experiences for our second-year veterinary students engaging them in activities despite COVID- 19 restrictions limiting face-to-face classes.

## Course structure and development

In January 2020, we developed a course in Evidence-Based Veterinary Medicine (EBVM) for the second-year students in a 6-year Bachelor of Veterinary Medicine program (BVM) at City University of Hong Kong. Prior to designing the course, we reviewed the underlying pedagogy for teaching veterinary medicine and EBVM using an outcome-based approach and active learning (3, 12), which promotes rote learning. Instead, we wanted the EBVM course to facilitate students' ability to use “Just-In-Time” information, with the teacher acting as a facilitator rather than the source of truth. The latter is a skill that is important to clinicians in practice (3).

The students had 1 year of general undergraduate sciences completed, which included a basic statistics course when they enrolled in our EBVM course. We had in-depth knowledge of their biostatistics background, so we were able to link concepts from this course and build on student's prior knowledge. We also understood which of the foundational concepts required further coverage within the EBVM course. Additionally, students were signposted at various points in both courses as to where the knowledge would be useful within the 6-year programme (for example the 5th and 6th year research projects). Finally, the learning outcomes in this course were reviewed to determine their alignment with the expected “Day One Competencies” of City University of Hong Kong's veterinary graduates.

The EBVM course was originally supposed to be delivered face-to-face, but due to the COVID 19 situation in Hong Kong in February 2020, it had to be delivered using online teaching within 3 weeks of the commencement of the first class. The second year of this course, which started in January 2021 (referred to as the second cohort) was completed face-to-face. We report the issues and modifications used for the two delivery methods when relevant.

The intended learning outcomes for this course included the following: (1) Describe the principles of evidence-based veterinary medicine; (2) Demonstrate critical appraisal of clinical research, including evaluating external and internal validity of studies, diagnostic test interpretation, measures of association, and bias analysis; and (3) Answer a clinical question in PICO format (Patient, Intervention, Comparison, Outcome) (13) using evidence from the literature (Table 1).

**Table 1:** Course intended learning outcomes, activities to teach these and assessment methods used in the evidence based veterinary medicine course described.

**Learning outcome**	**Activity and topics**	**Assessment**
Demonstrate understanding of the evidence-based veterinary medicine (EBVM) method including understanding study design, evaluating external and internal validity of studies, diagnostic test interpretation, measures of association and effect, and bias analysis	Lecture: Introduction to EBVM (Five steps of the EBVM approach: Ask, Acquire, Appraise, Apply, Assess)	1)Examination
	Lecture: Measuring clinical outcomes (types of clinical measurements, incidence, prevalence, and morbidity/mortality measures)	
	Lecture: Sampling (population sampling strategies commonly used in veterinary medicine practice/research)	
	Lecture: Study designs (introduction to various study types including cross-sectional, case-control, cohort, interventional)	
	Lecture: Measuring associations and effects (introduction to odds ratio, risk ratio, attributable risk and fraction, and evaluating statistical significance)	
	Lecture: Diagnosis and evaluation of diagnostic tests (concepts of screening and diagnostic test sensitivity, specificity, and predictive values)	
	Lecture: Bias detection, assessment, & control (identification of three types of bias using practical examples: selection, information, and confounding)	
Appraise clinical research critically	Lecture on critical appraisal of studies	2) Completion of assessment forms 3) Active participation in journal club meetings
	Journal club: case report/series	
	Journal club: cross sectional study	
	Journal club: case control study	
	Journal club: cohort study	
	Journal club: clinical trial	
Answer a clinical question using evidence from the literature	Lecture and library session: systematic review/search	4)Knowledge summary assignment
	knowledge summary (PICO) project (in and out of classroom activity)	

The course used several strategies to achieve these objectives (Table 1), including (1) traditional didactic lectures to provide basic information to students; (2) practical literature review and critical appraisal exercises (journal club); and 3) a knowledge summary which critically appraised a topic to answer a clinical question to teach students how to apply knowledge – an approach that has previously been described as “Scaffolded Active Learning” (1).

The course also used a variety of assessment methods (Table 1). We had traditional written exams with multiple choice and short answer questions to test knowledge. Students were also assessed when they reviewed papers with us (journal club) each week to practice extracting information and evaluating the quality of evidence in the papers. Lastly, we had a graded knowledge summary small group exercise, similar to that described in Steele et al. (14) that allowed us to assess whether the students could interpret and synthesize information from multiple sources to answer a clinical question. The latter was the ultimate goal of the course.

The course was structured in two parts. First, we taught general principles of epidemiology and evidence-based veterinary medicine, which covered topics such as how to formulate an answerable clinical question, conduct a systematic literature search, how to read peer-reviewed papers, how to analyze the strength, magnitude, and validity of the findings from different types of study designs (case report/series, cross-sectional, case-control, cohort, and clinical trials). The initial epidemiological concepts were taught in a didactic manner on zoom for both cohorts due to COVID restrictions. We had interactive questions during the lectures, but that was the limit of active learning for the initial component of the course. Students were assessed on these fundamental principles using a summative exam on the 6th and 8th week of the course for the first and second cohorts, respectively. The second part of the course consisted of the active learning through journal clubs and a group based knowledge summary activity.

### Knowledge summary assignment

Students were given their knowledge summary assignment after their summative written examination, which followed the last diadactic lecture on epidemiological concepts. This assignment consisted of conducting a review of a clinical question using the PICO framework. Groups of three to four students were assigned a PICO question to answer using published peer-reviewed literature. Each group had to follow the format prescribed by the Royal College of Veterinary Surgeons (RCVS) Knowledge (https://veterinaryevidence.org/index.php/ve/guidelines-for-authors) with the aim of publishing the reviews at the end of the course in their journal, Veterinary Evidence. This assignment enabled students to practice systematic search and critical appraisal of relevant literature to answer a clinically relevant question, and make a conclusion based on the information they had acquired. This knowledge summary exercise also helped them work as a team, and communicate their findings in written form, both of which are critical skills for veterinarians and are recognized as an essential part of a veterinarian's employability (15). To ensure that the students were progressing in a timely manner with this assessment, we had two “check-in” points. First, they had to provide us with their list of search terms and inclusion and exclusion criteria. This permitted us to set the students on the right track if they were initially off-target to ensure they had the right articles to read. We also had them provide us with the number of papers that met their criteria to make sure their searches had sufficient articles to review.

### Journal club (literature review)

During the second half of the course, journal clubs were initiated for students to actively learn how to critically appraise different types of studies. This activity consisted of six to eight student discussion groups supervised by either a graduate student or a faculty member. We rotated the group supervisor for each group every week. The use of graduate students during the small group discussions with undergraduate students served as a good opportunity for graduate students to practice the EBVM approach and gain experience in a leadership role managing small group discussions. The lack of time to devote to journal clubactivites has been identified by others as a limiting factor for incorporating this type of exercise in courses (16) despite its potential benefits on learning in medical programmes (17, 18). Using graduate students to assist in the management of the undergraduate BVM student journal club groups also addresses this issue and provided a learning experience for the graduate students.

### Graduate student training in critical appraisals

We met with the graduate students involved in this project every week prior to the class discussion groups. Prior to each weekly meeting, we provided the undergraduate and graduate students with an article and a question sheet to help guide their reading (see Supplemental Information to see the sheet provided to the second cohort, which was modified from the first version). We specifically selected articles that illustrated different concepts learned in the first half of the semester. For example, each paper had a different study design (case series, cross-sectional, case-control, cohort, and clinical trials). We also purposefully selected articles that demonstrated different types of bias (with examples of selection bias, information bias, and confounding bias).

In meetings between faculty and graduate students, we asked different graduate students to lead the discussion groups every week in reviewing the papers using the questions provided. Here, we ensured that all students left the meeting understanding the main discussion points that were important to the specific paper being reviewed so they could ensure these were discussed in the undergraduate sessions. We reviewed some scenarios with the graduate student on how to probe students if they did not understand or bring up certain key topics pertinent to the individual papers.

### Undergraduate student literature review sessions (journal club)

Each student was provided with a paper to read each week for 5 weeks and a list of questions for students to address in order to assist with the group discussion (refered to as the question sheet and listed in the Supplemental Materials). Prior to the discussion period, students were asked to hand a copy of their responses to the questions. This was used to ensure the students read the paper prior to the class. It was included as part of their final assessment, but it was not graded; however, the faculty reviewed the answers to gauge the level of student understanding. Each student also was asked to lead and act as secretary for at least one session, making use of peer-to-peer learning, which has been demonstrated to improve skill levels (19, 20). At the end of the session, the group had 24 h to complete a revised question sheet and submit their answers for a graded group assignment mark. To encourage participation in the discussion students were also given a grade on participation as part of their mark each week. This mark was determined by the supervisor in the group and was a binary score (0/1). Students received this mark if they participated regardless of the content of their answers unless their contribution was disruptive.

Each group of undergraduate students was assigned a group supervisor (graduate student or faculty). The role of the supervisor was to ensure the student group discussion stayed on track and that all questions in the guide were discussed. If the students did not understand a specific topic, it was the role of the supervisors to probe the group with questions to help students discover the learning objectives. If this did not work after a few questions, the supervisor intervened and reviewed the concept with the group.

## Discussion

The first cohort of students consisted of 29 students, and the second cohort had 23 students.

### Didactic lecture content

The topics covered in this course included: introduction to evidence-based veterinary medicine, causation verses associations, measures of clinical outcomes, clinical decision making and the role of economics, sampling, study design, measuring effect and statistical significance, diagnostic test evaluation, bias in clinical research, and systematic review of the literature. The average score on the written exam for the first and second cohort of students was 86.5 and 88.3%, respectively. We increased the didactic lectures in the second year from 15 to 21 h to address the contents in more depth.

### Undergraduate literature review sessions

We reviewed 16 papers in the first year and reduced this to five in the second year to ensure that the students had sufficient time to read and understand the literature. There has been research suggesting that students who feel their workload is too high are more likely to adopt a surface learning approach (4), where they study only the necessary information to obtain a certain grade, defeating our learning objective. We also reduced the number of papers in the second year to accommodate the increase in didactic lectures. The types of papers covered in the literature review component of this course included at least one case series, cross-sectional, case-control, cohort, and clinical trial studies. Another change that we made in the second year was to streamline the question sheet used in our review sessions to focus the discussion about the papers. Lastly, in the second year, we required students to provide us with a copy of their answers to these questions prior to the group meeting to ensure they had thought about the paper before the group discussion. Anecdotally, this seemed to facilitate the discussion between students. Pedagogically, this can be likened to the use of a flipped-classroom approach, where students are required to familiarized themselves with the basic principles of a topic outside the classroom, and during class engage in applications of concepts with the guidance of the instructor. This teaching strategy increases student engagement and improves learning outcomes when used in a variety of settings in veterinary medicine (21, 22). The first cohort of students met online to discuss the papers, but the second cohort had face-to face meetings. The latter seemed to increase the level of engagement.

Students commented on their evaluations that they found this exercise difficult, especially at the beginning of the course, but eventually improved in their critical assessment of research studies. We believe our study question sheet helped guide them through the papers in a systematic manner, which helped train them over time. Graduate students also seemed to improve in their ability to identify key points in papers as the course progressed. Journal clubs or literature review sessions in professional medical schools have been reported to help student knowledge (17). These authors evaluated the use of journal club for dental students at the University of Glasgow and found a positive effect in knowledge and confidence scores, but attendance to their club was optional and it was only attended by a small fraction of students. In our course, participation was graded, which likely contributed to excellent attendance rates.

### PICO knowledge summary analysis

Twelve different PICO knowledge summaries were conducted the first year this course was offered as each group of students had to answer their own clinical question. These questions were selected from the Veterinary Evidence website (https://veterinaryevidence.org/index.php/ve/clinical-queries) or developed by locally based clinicians to address a practice-based knowledge gap specific to the region. This proved very onerous to grade, so for the second cohort, we limited the number of questions to two (one per faculty), and we assigned each question to three groups of students. At the end of the second cohort, we compiled the results of all the groups addressing the same PICO and submitted the results for publication to Veterinary Evidence. One paper has been published (23), and the other is under review.

### Overall learning experience

Students provide annual feedback on taught courses via a questionnaire consisting of 10 questions marked on a seven point scale (see Supplemental Information). The overall score for this course was 6.80/7 and 6.24/7 for the first and second cohorts, respectively. The average score for all University courses in these two years was 5.89/7 and 5.76/7, respectively. In the first year, we had a 34% response rate, and in the second year, a 91% response rate to the survey. Some comments from students included “This is overall an awesome course and well–executed VM course. I particularly enjoy the knowledge summary assignment that allows us a chance to publish, it makes me really think I am learning medicine” and “Even though it is difficult, it provides really helpful skills for the future! The professors were very helpful and always answered our emails when doing the PICO assignment.” suggesting they enjoyed the learning experience and found it valuable.

The PICO exercise provided an interactive style of learning, which has been shown to enable students to develop active learning skills, encourage deep learning through engagement, and develop students as lifelong learners (4, 24). Group work and the novelty of the method of teaching presented here are recognized challenges (10). However, group work provides numerous benefits and supervision by more experienced near-peers allows students to lead their own learning, and improves the knowledge and understanding of the near-peers who are guiding them (25).

## Conclusion

There are several other examples of active learning activities for EBVM courses (10, 11, 14, 16, 26–28). In the course we describe in this perspective piece, we incorporated several suggestions from these papers, alongside established pedagogical approaches for veterinary medicine (3). Our course serves as an example of implementing active learning to achieve and demonstrate deep learning in an EBVM course. The success of the course is reflected in the students' published knowledge summaries, which demonstrate the objective of the course to use evidence to make clinical decisions. It is recognized that deeper evaluation of the students' learning should be assessed objectively and this represents a starting point for future work. The use of several teaching and assessment methods made this course engaging for everyone involved. It is recognized that the use of novel approaches, including the use of clinical cases and EBVM approaches, improves student engagement (27). Designing and implementing this course took substantially more time, and effort than simply providing didactic lectures on epidemiology, but it may be a good example of how to get students engaged in a field that is usually not well appreciated by undergraduate BVM students.

## Data availability statement

The original contributions presented in the study are included in the article/supplementary material, further inquiries can be directed to the corresponding author/s.

## Ethics statement

Ethical review and approval was not required for the study on human participants in accordance with the Local Legislation and Institutional requirements. Written informed consent for participation was not required for this study in accordance with the National Legislation and the Institutional requirements.

## Author contributions

SR and SS-H designed and implemented the initial course. SS-H and ON developed new materials, improved, and refined the format of the course. ON is the current course leader. SR, ON, RP, and SS-H reviewed pedagogical approaches associated with active learning and EBVM. SS-H wrote the first draft of the manuscript. SR, ON, and RP edited the document. All authors contributed to the article and approved the submitted version.

## Conflict of interest

SR was employed by Ag Research Limited.

The remaining authors declare that the research was conducted in the absence of any commercial or financial relationships that could be construed as a potential conflict of interest.

## Publisher's note

All claims expressed in this article are solely those of the authors and do not necessarily represent those of their affiliated organizations, or those of the publisher, the editors and the reviewers. Any product that may be evaluated in this article, or claim that may be made by its manufacturer, is not guaranteed or endorsed by the publisher.
