# Supplementary material for: Active learning for an evidence-based veterinary medicine course during COVID-19
Source: Front Vet Sci. 2022 Jul 22;9:953687. doi: 10.3389/fvets.2022.953687 (PMC9353066; doi:10.3389/fvets.2022.953687)
Supplement: Supplementary file 1 [file Data_Sheet_1.docx]

**Supplemental information**

**“Question Sheet for the review of studies”**

If a question is not relevant to the study type, please indicate ‘NA’

| **Introduction** | |
| --- | --- |
| What is/are the objective/s of the study? | Describe |
| **Materials & methods** | |
| What is the ‘study type’? | Study type (choose from the key)  Briefly, explain your choice |
| What is the study population? | Define |
| What is the target population? | Define |
| How were the study subjects selected? | Sampling strategy (choose from the key)  Briefly, explain your choice |
| What are the key ‘inclusion’ and ‘exclusion’ criteria in selecting the subjects? | Inclusion criteria:  Exclusion criteria: |
| What is the sample size? | ‘n’ |
| What is/are ‘the outcome/s of interest’?  (Explain how each one is measured) | List all ‘outcomes’ measured in this study |
| What is/are the predictor(s)/exposure(s) of interest?  (Explain how each one is measured) | List all ‘predictors’ measured in this study |
| What are the statistical tests used? | List |
| ‘Study type’-specific questions |  |
| 1. Case report/series |  |
| What is the ‘case definition’? |  |
| 1. Cross-sectional study |  |
| What is/are the ‘comparison group/s’? |  |
| 1. Case-control study |  |
| What is the ‘case definition’? |  |
| Define the control group(s) |  |
| 1. Cohort study |  |
| What is the ‘exposure’ of interest? |  |
| What is the ‘unexposed’ comparison group? |  |
| How long is the duration of study/follow-up? |  |
| Is the outcome measured in a repeated manner? | Yes/No/Unclear |
| 1. RCTs |  |
| What is the ‘intervention’ of interest? |  |
| What is the control group(s)? |  |
| Was ‘randomization’ implemented? How? | Yes/No/Unclear |
| Was ‘blinding’ implemented? How? | Yes (one, two, three sided)/No/Unclear |
| Was ‘ethic approval’ sought? | Yes/No/Unclear |
| **Results** | |
| What are the key findings? | 1-5 in bullet points |
| What are the ‘measures of association and/or effect’ used to compare the study groups? | (OR, RR, IRR; AR, AF, PAR, AFp)  Choose and express their ‘magnitude’ = … |
| Are the specified measures statistically significant? | Yes/No/Unclear |
| **Discussion & conclusions** | |
| List the key conclusions of the study | 1-3 bullet points |

**“Critical Appraisal” of the study**

| **Introduction** | |
| --- | --- |
| What is the justification for conducting this study? | Provide 1-2 key points |
| **Materials & methods** | |
| Is the ‘study type’ suitable for addressing the objectives/questions of interest? | Yes/No/To some extent  Provide 1-2 main reasons for your answer |
| Is the ‘sampling strategy’ suitable for addressing the objectives/questions of interest? | Yes/No/To some extent  Provide 1-2 main reasons for your answer |
| Are the study groups ‘comparable’? | Yes/No/to some extent  Briefly, justify your answer |
| Are the statistical test/s used appropriate? | Yes/No (briefly explain) |
| Are the ‘measures of association or effect’ used appropriately for the type of study? | Yes/No (briefly explain) |
| **Results** | |
| Are the basic data adequately described? | Yes/No/To some extent  Briefly, explain your answer |
| Are the table/s and graph/s useful? | Yes/No/To some extent  Briefly, explain your answer |
| Do the ‘numbers’ add up? | Yes/No |
| **Discussion & conclusions** | |
| Does the discussion reflect the results? | Yes/No/To some extent  Briefly, explain your answer |
| If the authors are claiming ‘causality’, is there sufficient evidence? | Yes/No (briefly explain) |
| Is there evidence of selection bias? | Yes/No (briefly explain) |
| Is there evidence of information bias? | Yes/No (briefly explain) |
| Is there evidence of confounding bias? | Yes/No (briefly explain) |
| Can the study results be applied more widely to other populations and to the intended target population? (external validity) | Yes/No (briefly explain) |
| Are the main results consistent with other evidence? | Yes/No/To some extent (briefly explain) |
| What are the practical implications of the study? | 1-3 key points |

**Keys for ‘study type’ and ‘sampling strategy’**


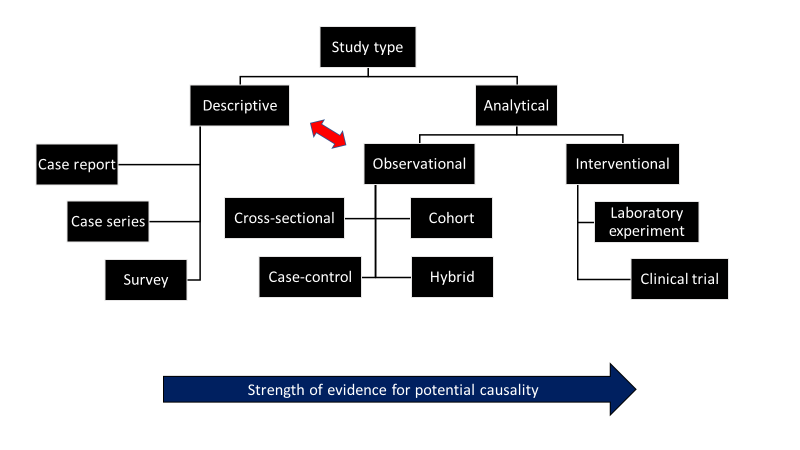


…………………………………………………………………………………………………………………………………………………………


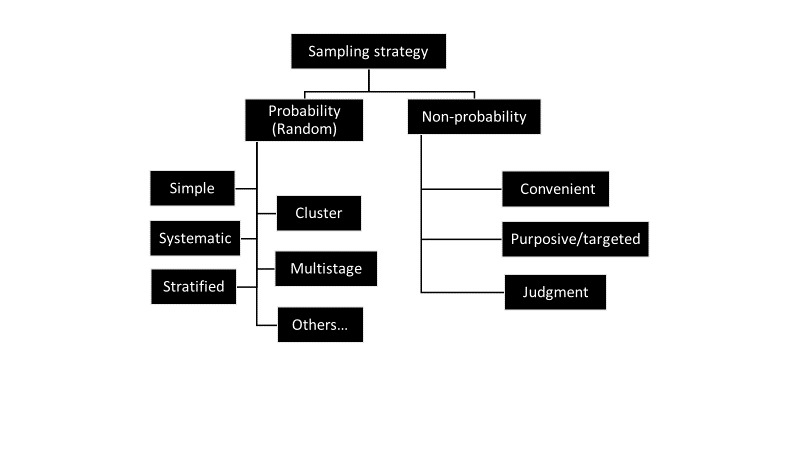


**The Teaching and Learning Questionnaire (TLQ)** **provided to the students to evaluate the instructors and courses**

The questionnaire contains 2 types of questions. Responses to the first 10 questions, which cover teaching, learning experience, and an overall evaluation of the course are on a scale of 1 (strongly disagree ) to 7 (strongly agree). There are two open-ended questions with free response.

Likert scale Questions

1. The teacher was well prepared for class.
2. The teacher’s instruction and explanations were clear.
3. The teacher provided useful feedback and comments.
4. The teacher was helpful.
5. The teacher used English as the medium of instruction throughout the course.
6. I found the learning experience well designed.
7. I was encouraged to be creative/innovative.
8. I was encouraged to think critically.
9. I found the course difficult.
10. Overall, I consider the learning experience provided by the teacher in this course as valuable.

**Open-ended questions**

1. What were the best aspects of the course?
2. What aspects of the course were most in need of improvement?
